# Study of preserving the PTCD tube after laparoscopic pancreaticoduodenectomy

**DOI:** 10.1097/MD.0000000000032813

**Published:** 2023-02-03

**Authors:** Haojun Wu, Xia Zeng, Ying Liang, Bei Li, Liping Chen

**Affiliations:** a Department of Biliary Surgery, West China Hospital, Sichuan University, Chengdu, Sichuan, China; b Department of Ultrasound, Shangjin Nanfu Hosptial, Chengdu, Sichuan, China.

**Keywords:** laparoscopic pancreaticoduodenectomy, percutaneous transhepatic cholangial drainage

## Abstract

Severe jaundice patients undergoing laparoscopic pancreaticoduodenectomy (LPD) tend to choose percutaneous transhepatic cholangial drainage (PTCD) for preoperative biliary drainage. However, there are few studies on whether to preserve PTCD drainage tubes after surgery. This study tentatively discusses that jaundice patients preserving the PTCD tube have similar postoperative recovery to that in ordinary patients undergoing LPD. We retrospectively reviewed 46 patients who underwent LPD between June 2019 and April 2022 at our department. They were divided into a drainage group with 16 patients and a normal group with 30 patients according to whether PTCD was performed. Patient demographics, perioperative data, and postoperative outcomes were observed and counted. The preoperative total bilirubin in the drainage group was significantly higher than that in the normal group. There was no significant difference in age, body mass index, American Society of Anesthesiologists grade, hemoglobin, albumin, operation time, postoperative hospital stay, or total complication rate between the 2 groups. The PTCD tube was preserved in all 16 patients after the operation, and only 1 patient (6.3%) developed PTCD-related postoperative complications, which were dislocations. It is safe and effective to choose PTCD to reduce jaundice before surgery and preserve PTCD tubes after surgery for moderate and severe jaundice patients who plan to undergo standardized and streamlined LPD. These patients achieve similar postoperative recovery of LPD as no-drainage patients.

## 1. Introduction

Laparoscopic pancreaticoduodenectomy (LPD), which was first reported in 1994,^[1]^ is considered one of the most challenging operations. Currently, with the progress of surgical technology and postoperative nursing, the number of hepatobiliary and pancreatic surgeons who master LPD is increasing rapidly all over the world, and experienced centers have been able to control the incidence of complications and postoperative mortality to an acceptable level.^[2]^ An increasing number of clinical studies have confirmed the safety, feasibility, and prognosis of LPD similar to open pancreaticoduodenectomy, and its indications are expanding.^[3–6]^

For patients with obstructive jaundice before surgery, although the guidelines of China, Europe, and America do not recommend routine preoperative biliary drainage, many researchers still believe that preoperative biliary drainage is beneficial for patients with severe jaundice and tend to choose percutaneous transhepatic cholangial drainage (PTCD).^[7]^ However, there are few studies on whether to preserve PTCD drainage tubes after surgery. This paper retrospectively observed the clinical data of 46 LPD patients in our center from June 2019 to February 2022 and preliminarily summarized its safety and effectiveness. The postoperative recovery of jaundice patients preserving the PTCD tube was analyzed and compared with that of ordinary patients.

## 2. Methods

### 2.1. Patients

From June 2019 to April 2022, 46 patients who underwent LPD for variable diagnoses at our department were retrospectively collected. Inclusion criteria: the patient underwent standard LPD surgery, and clinical data were complete. Exclusion criteria: change the operation mode during operation, change to palliative gastrointestinal anastomosis, biliary-intestinal anastomosis, etc; and vascular resection and reconstruction were performed during the operation. Among them, 16 patients underwent PTCD to reduce jaundice before the operation, and the PTCD drainage tube was reserved after LPD, which was divided into the drainage group. The other 30 patients who underwent routine LPD were divided into the normal group. This study was approved by the institutional review board of West China Hospital 2022 (712).

### 2.2. PTCD operation method

1. Instruments and equipment

The instrument used was a Philips IU22 color Doppler ultrasound diagnostic instrument, and the probe frequency was 2.5 to 5.0 MHz, the puncture needle was a 17G coaxial puncture needle, 8.5 F drainage tube (bud pigtail drainage cannula with curled front end and side hole), super sliding guide wire (0.035 in diameter, soft J-shaped front end), and sharp blade (No. 11 blade).

1. Seldinger technology

All patients chose the left branch as the puncture bile duct. Patients were placed in the supine position. Routine iodophor disinfection of skin was performed after ultrasonic determination of the needle insertion route. Spread sterile hole towels. Local anesthesia with 2% lidocaine injection reached the liver capsule. An incision of approximately 2 mm was made at the skin location with a sharp knife. Under the guidance of ultrasound, the 17 G coaxial puncture needle was quickly pierced into the preselected target bile duct by a bare-hand puncture. Pull out the needle core. After the bile flowed out with a syringe, the guide wire was introduced into the bile duct. The direction of the guide wire was appropriately adjusted and placed in the common bile duct. After confirming that the guide wire was in place, a flat-headed metal needle sheath was used to place the drainage tube along the guide wire. After supporting the catheter with a support tube, insert the drainage tube along the guide wire (the expansion tube at the hard head end expands the needle path step by step along the guide wire and then insert the drainage tube). After bile drainage was unobstructed, the sliding line of the drainage tube was pulled to make the end of the drainage tube curl in a “pig tail” shape, the drainage bag was connected, the drainage tube was fixed on the skin, and the tube was covered and fixed with sterile gauze.

### 2.3. LPD operation method

**Figure 1.**
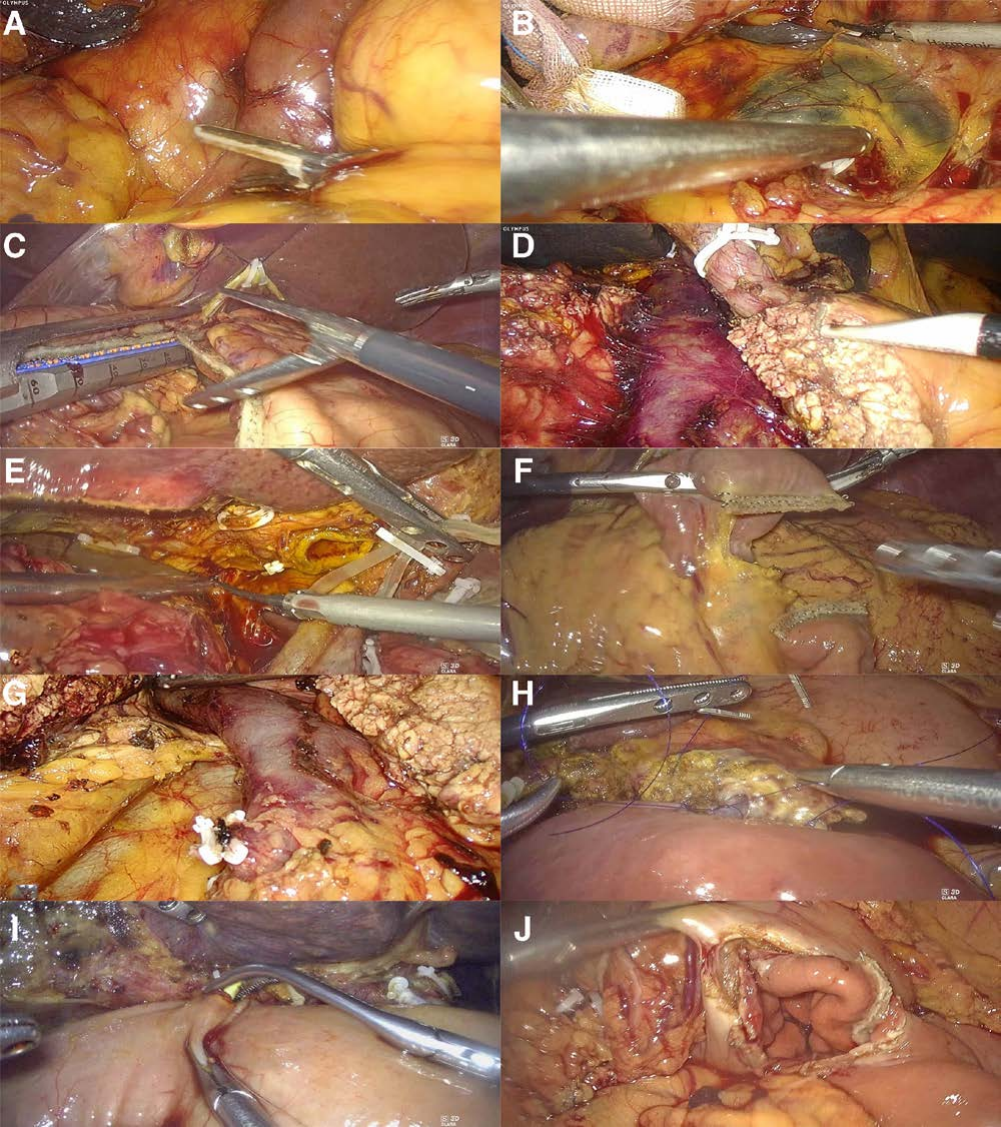
Streamlined and modular surgical process for LPD. (A) Descent of the hepatic flexure of the colon. (B) Kocker procedure. (C) Amputation of the distal stomach. (D) Amputation of the pancreas. (E) Amputation of the hepatoduodenal ligament and bile duct. (F) Amputation of the jejunum. (G) Amputation of the uncinate process of the pancreas. (H) Pancreaticoenterostomy. (I) Biliary enterostomy. (J) Gastrointestinal anastomosis. LPD = laparoscopic pancreaticoduodenectomy.

**Figure 2.**
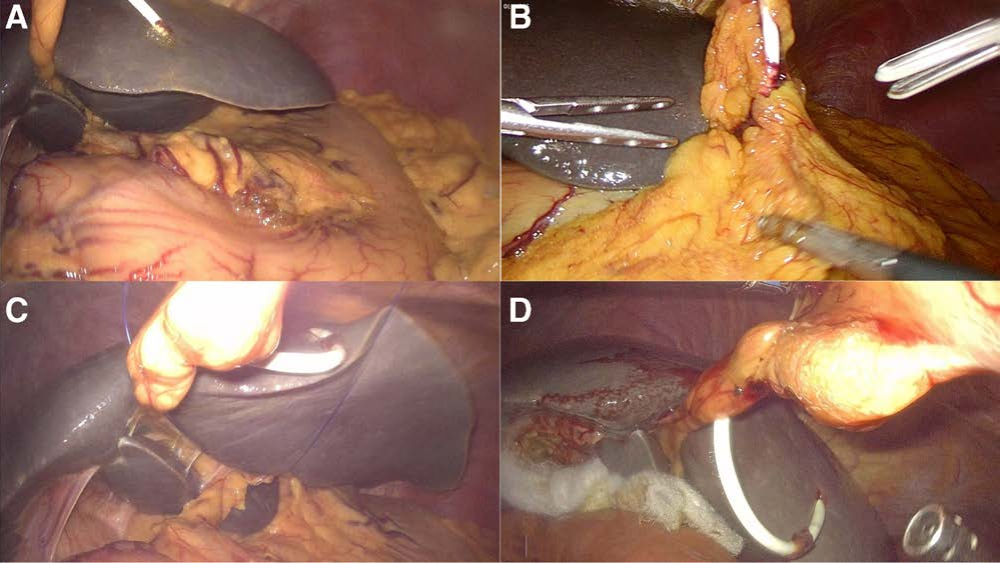
PTCD tube at the beginning and end of the operation. (A and B) Beginning of operation. (C) During the operation. (D) End of operation. PTCD = percutaneous transhepatic cholangial drainage.

Our department adopts a streamlined and modular surgical process, as shown in Figure 1. The intraoperative image of the PTCD tube is shown in Figure 2.

### 2.4. Data collection and variable definitions

If the total bilirubin is >200 μmol/L in LPD patients in our department, PTCD drainage is used to reduce jaundice, and the endpoint of biliary drainage is generally <200 μmol/L. In this study, demographic and periodic data were retrospectively reviewed. In general, postoperative morbidity was classified according to the Clavien–Dindo classification.^[8]^ Complications such as postoperative pancreatic fistula,^[9]^ post pancreatic hemorrhage (PPH),^[10]^ and delayed gastronic emptying^[11]^ were defined by concern and guidelines. In addition, PTCD-related complications, such as blockage and shedding of the drainage tube, cholangitis, and biliary hemorrhage, were also counted.

### 2.5. Statistical analysis

Continuous variables are expressed as the mean with standard deviation or median with interquartile range. Categorical data are presented as numbers with percentages. The significance of continuous data was tested using Student *t* test or the Mann–Whitney *U* test. The significance of categorical data was tested using the *x*^2^ test or Fisher exact test.

## 3. Results

### 3.1. Patient demographics

A total of 46 patients underwent LPD, 16 in the drainage group and 30 in the normal group. As presented in Table 1, the patients included 20 men (43.5%) and 26 women (56.5%), with a median age of 58 years. Body mass index was 21.6 kg/m^2^. Most patients (78.3%) were categorized as American Society of Anesthesiologists grade II; the remaining patients (21.7%) were categorized as American Society of Anesthesiologists grade III, and there was no significant difference in the patients’ characteristics between the drainage group and the normal group. The median preoperative hemoglobin (124.5 g/L) and median albumin (38.5 g/L) levels were normal, with no significant difference between the 2 groups, whereas the median total bilirubin level (90.5 μmol/L) was significantly elevated and was higher in the drainage group (*P* < .001). In this study, there was also no significant difference in the patients’ pathological diagnoses between the 2 groups. Pancreatic cancer (26.1%) and duodenal cancer/duodenal papillary cancer (21.7%) were the most common indications, followed by distal biliary duct cancer (17.4%) and ampullary cancer (17.4%). Unilateral drainage was performed in all patients in the drainage group, of which 14 (87.5%) had left hepatic duct drainage. The highest median total bilirubin before PTCD puncture was 296.1 (259.4–377.6) μmol/L, and no complications related to PTCD above Clavien–Dindo II occurred during biliary drainage.

**Table 1:** Demographic data of patients.

	Total: mean (SD)/n (%)/median (Q1–Q3)	The drainage group: mean (SD)/n (%)/median (Q1–Q3)	The normal group: mean (SD)/n (%)/median (Q1–Q3)	*P* value
No. of patients	46	16	30	
Sex (male)	20 (43.5%)	9 (56.2%)	11 (36.7%)	.064†
Age (yr)	58 (50.5–67.3)	59 (50.3–69)	58 (50.3–67.3)	.926†
BMI (kg/m^2^)	21.6 (20.5–24.1)	23.2 (20.7–24.5)	22.3 (19.7–24)	.288†
ASA grade				
II	36 (78.3%)	11 (68.7%)	25 (83.3%)	.283†
III	10 (21.7%)	5 (31.3%)	5 (16.7%)	.283†
Hemoglobin (g/L)	124.5 (111.8–138.3)	122 (113–136.8)	129.5 (110.8–138.5)	.628*
Total bilirubin (μmol/L)	90.5 (18.7–168.4)	132 (96.2–213)	41.9 (11.5–129)	**<.001** *
Albumin (g/L)	38.5 (34.4–41.7)	38.8 (34.4–41.9)	37.6 (34.4–41.8)	.628*
Diagnosis				
Duodenum cancer or duodenal papillary cancer	10 (21.7%)	1 (6.3%)	9 (30%)	.130†
Pancreatic cancer	12 (26.1%)	7 (43.8%)	5 (16.7%)	.077†
Distal biliary duct cancer	8 (17.4%)	3 (18.8%)	5 (16.7%)	>.999†
Ampullary cancer	8 (17.4%)	4 (25%)	4 (13.3%)	.421†
Chronic pancreatitis	2 (4.3%)	0	2 (6.7%)	.536†
Pancreatic cystic tumors	2 (4.3%)	0	2 (6.7%)	.536†
Others	4 (8.7%)	1 (6.3%)	3 (10%)	>.999†

Bold value indicates statistical significance (*P* < .05).

Others include intraductal papillary mucinous neoplasm, serous cystic neoplasms, mucinous cystic neoplasms, pancreatic neuroendocrine tumors.

ASA = American Society of Anesthesiologists, BMI = body mass index, SD = standard deviation.

* Mann–Whitney *U* test. †Fisher exact test.

### 3.2. Perioperative data and postoperative outcomes

As presented in Table 2, there were no significant differences in the operative time (drainage group 331 [281–387] min vs normal group 298 [260–371] min) or the postoperative hospital stay (drainage group 10 [8–11] days vs normal group 10 [9–14] days). Tissue adhesion was observed in 6 cases (37.5%) of PTCD puncture sites during the drainage group operation, of which 2 cases (12.5%) formed intact sinus tracts. After opening the common bile duct, a puncture tube was found in 9 cases (56%). Old blood clots in the bile duct were found in 2 cases (12.5%). One case (6.2%) was sutured and strengthened during the operation, and no PTCD tube slipped during the operation. There was no reoperation in the 2 groups; 90-day mortality was 3, 1 in the drainage group and 2 in the normal group. There was no significant difference in total postoperative complications between the 2 groups according to the Clavien–Dindo classification (drainage group 5 [31.3%] vs normal group 13 [43.3%]). The incidence of PPH and bile leakage in the drainage group was 0, but the difference was not statistically significant. PTCD tube drainage was unobstructed in all patients during postoperative hospitalization, and PTCD dislocation occurred in 1 patient (6.3%) during postoperative hospitalization, no postoperative PTCD-related complications, such as obstruction, bile duct hemorrhage, or cholangitis, occurred. There were 13 patients (81.3%) discharged with Only PTCD tube, and 5 patients (31.3%) removed PTCD tube within 1 month.

**Table 2:** Perioperative data.

	Total: mean (SD)/n (%)/median (Q1–Q3)	The drainage group: mean (SD)/n (%)/median (Q1–Q3)	The normal group: mean (SD)/n (%)/median (Q1–Q3)	*P* value
No. of patients	46	16	30	
Operative time (min)	319 (264–382)	331 (281–387)	298 (260–371)	.362*
Postoperative hospital stay (d)	10 (8–12)	10 (8–11)	10 (9–14)	.150*
Reoperation	0	0	0	
Readmission within 30 d	3 (6.5%)	0	3 (10%)	.542†
30-d mortality	2 (4.3%)	0	2 (6.7%)	.536†
90-d mortality	3 (6.5%)	1 (6.3%)	2 (6.7%)	>.999†
Complications	18 (39.1%)	5 (31.3%)	13 (43.3%)	.533†
Grade I/II	14 (30.4%)	4 (25%)	10 (33.3%)	.739†
Grade III/IV/V	4 (8.7%)	1 (6.3%)	3 (10%)	>.999†
Complications by category				
POPF	9 (19.6%)	3 (18.8%)	6 (20%)	>.999†
A	7 (15.2%)	3 (18.8%)	4 (13.3%)	.681†
B	1 (2.2%)	0	1 (3.3%)	>.999†
C	1 (2.2%)	0	1 (3.3%)	>.999†
Bile leakage	4 (8.7%)	0	4 (13.3%)	.282†
DGE	0	0	0	
VTE	0	0	0	
PPH	1 (2.2%)	0	1 (3.3%)	>.999†
Gastrointestinal bleeding	9 (19.6%)	4 (25%)	5 (16.7%)	.698†
SSI	3 (6.5%)	1 (6.3%)	2 (6.7%)	>.999†

Bold value indicates statistical significance (*P* < .05).

DGE = delayed gastric emptying, POPF = postoperative pancreatic fistula, PPH = postpancreatectomy hemorrhage, SD = standard deviation, SSI = surgical site infection, VTE = venous thromboembolism.

* Mann–Whitney *U* test.

† Fisher exact test.

## 4. Discussion

In 2010, Van der Gaag et al^[12]^ suggested that routine preoperative biliary drainage in patients with pancreatic head cancer with total bilirubin between 40 and 250 μmol/L is not recommended. Since then, the indications and goals of preoperative biliary drainage in patients with periampullary tumors complicated with obstructive jaundice have been controversial. To date, the guidelines of China, Europe, and America do not recommend routine preoperative biliary drainage. Some scholars believe that preoperative biliary drainage does not reduce the mortality and complication rate but prolongs the hospitalization time and may even increase the risk of postoperative infection.^[13,14]^ Some studies also show that a high serum bilirubin level before surgery may be an independent predictor of postoperative complications, which confirms the significance of preoperative biliary drainage.^[15,16]^ Clinically, some doctors still tend to reduce jaundice before surgery for patients with severe jaundice. The highest median bilirubin of the drainage group was 296.1 (259.4, 377.6) μmol/L, and the median total bilirubin of the drainage group before operation was 179.8 (165.7, 185.6) μmol/L, which was significantly different from that of the normal group. In the drainage group, the most time to reduce jaundice was within 2 weeks (11 cases, 68.9%). The endpoint of biliary drainage is generally that bilirubin drops <200 μmol/L, which will also be comprehensively evaluated in combination with the characteristics of bile drainage, bilirubin decline rate, and general improvement.

Because there are many methods of preoperative biliary drainage, there are many studies on these different methods. Recent studies have shown that for LPD or open pancreaticoduodenectomy patients, more scholars believe that the safety and effectiveness of PTCD will be better than those of endoscopic retrograde cholangiopancreatography.^[17,18]^ Our center also believes that placement of a nasobiliary duct or temporary stent tube through endoscopic retrograde cholangiopancreatography will obviously aggravate inflammatory edema in the hepatoduodenal ligament area and affect the surgical operation. Therefore, in this study, all patients in the drainage group were treated with PTCD to reduce jaundice before the operation. Ultrasound can evaluate the diameter of the dilated intrahepatic bile duct and whether there are tumors and large blood vessels in the puncture path, dynamically observe the puncture process of the puncture needle in the whole process, and accurately enter the bile duct cavity, which is simple and safe to perform. Therefore, ultrasound-guided PTCD has been widely used in the clinic. When biliary obstruction is caused by low obstruction, both the left and right bile ducts can be used as target bile ducts. Because the left bile duct has no rib occlusion, is far away from the thoracic cavity, and is close to the isolated surface, which is more conducive to operation, the left bile duct is often selected as the target puncture point. In this study, PTCD puncture through the left bile duct was also significantly greater than that through the right bile duct (87.5% located in the left bile duct).

LPD is different from open pancreaticoduodenectomy, which does not pull the thorax or turn the liver during operation, and the operation hardly causes the PTCD tube to shift and fall off. It should be noted during the operation that the end of the PTCD tube may be located near the cross-section of the bile duct, so the anterior wall of the bile duct should be severed and explored during the operation, and attention should be given to protection. If the PTCD drainage tube can be seen at the broken end of the bile duct, the sliding line of the PTCD tube should be cut to avoid missewing during biliary-intestinal anastomosis, which will affect the removal of the PTCD tube after the operation. Previous literature reported that the incidence of PTCD-related complications was 3 to 30%,^[19]^ and drainage tube blockage (7.6%), dislocation (5.7%), and cholangitis (3.7%) were the most common associated complications.^[20]^ In this study, PTCD was performed during the operation in the drainage group, and 1 case was reinforced again during the operation. There were 2 cases of sinus formation, all of which had a long time to reduce jaundice. One case (6.3%) had PTCD prolapse after the operation, and the drainage changed from bile to ascites without abdominal bleeding and bile leakage. The literature reported that the incidence of biliary bleeding after PTCD was 4.6%.^[21]^ In this study, the incidence of gastrointestinal bleeding in the drainage group was 25%, all of which showed black stool and decreased hemoglobin. During hospitalization, the Clavien–Dindo grade did not exceed Grade II. One case recurred gastrointestinal bleeding after discharge and did not see a doctor in time. On the 41st day after the operation, the patient returned to the emergency department again and died because the rescue was ineffective. None of the patients underwent postoperative gastroscopy for gastrointestinal bleeding, so it was impossible to determine whether it was caused by biliary bleeding, gastrointestinal ulcer, or anastomotic complications. The author thinks that the incidence of complications of PTCD drainage tubes may be lower before and after operation in the center where PTCD is carried out earlier and the operation volume is large, and it is safe to keep drainage tubes after operation.

The total postoperative complication rate in this study was 39.1%, similar to the results reported in other recent LPD studies,^[22,23]^ and there was no significant difference between the 2 groups. No delayed gastronic emptying or venous thromboembolism occurred in the 2 groups. There was no significant difference in the incidence of pancreatic leakage, bile leakage, abdominal bleeding, gastrointestinal bleeding, or incision infection, and there was no bile leakage or PPH in the drainage group. In this study, the postoperative hospital stay was 10 (8–12) days, and there was no significant difference between the drainage group and the normal group. PTCD tubes were removed within 1 month after the operation in 5 cases in the drainage group, and the other PTCD tubes were removed more than 1 month after the operation. No PTCD-related complications, such as biliary peritonitis, biliary hemorrhage, or abdominal hemorrhage infection, were found during follow-up. We believe that preoperatively performing PTCD to reduce jaundice and postoperatively preserving the PTCD tube can weaken the influence of moderate and severe jaundice on the complication rate and postoperative hospitalization days of LPD patients and will not significantly increase the postoperative risk of LPD. This is similar to the results of some studies with a positive attitude toward PTCD. For example, El Nakeeb et al^[24]^ believe that preoperative drainage can significantly reduce the incidence of postoperative pancreatic fistula, biliary fistula, and other complications. Earlier, Sewnmh et al^[25]^ reported that the positive effects of PTCD drainage tubes on the body may be concealed by their negative effects in open surgery, so minimally invasive surgery and continuous improvement and optimization of PTCD puncture materials and techniques may greatly reduce the negative effects of PTCD and make the positive effects of preserving PTCD tubes more prominent after surgery.

The major limitations of this study are its retrospective nature and short follow-up. The sample size was also small. The efficacy of preoperative PTCD tubing could not be evaluated statistically more accurately in this study design because the bilirubin values of the 2 groups were different. Another limitation is that this was a single-center study. Due to the uncertain role of preserving the PTCD tube after LPD, a prospective, multicenter, randomized controlled trial of whether preserving the PTCD tube in LPD should be conducted.

In summary, it is safe and effective to choose PTCD to reduce jaundice before surgery and keep PTCD tubes after surgery for patients with moderate and severe jaundice who plan to undergo standardized and streamlined LPD. The incidence of complications and postoperative hospitalization days were similar to those of patients with simple LPD. Follow-up high-quality prospective randomized controlled trials are needed to determine whether PTCD tube retention truly benefits these patients after surgery.

## Author contributions

**Conceptualization:** Bei Li, Liping Chen.

**Data curation:** Haojun Wu, Ying Liang.

**Formal analysis:** Xia Zeng, Ying Liang.

**Methodology:** Haojun Wu.

**Project administration:** Bei Li, Liping Chen.

**Resources:** Xia Zeng, Bei Li.

**Supervision:** Liping Chen.

**Validation:** Haojun Wu, Xia Zeng, Ying Liang, Liping Chen.

**Visualization:** Haojun Wu, Xia Zeng.

**Writing – original draft:** Haojun Wu, Xia Zeng.

**Writing – review & editing:** Bei Li, Liping Chen.
